# Structures of B-Lymphotropic Polyomavirus VP1 in Complex with Oligosaccharide Ligands

**DOI:** 10.1371/journal.ppat.1003714

**Published:** 2013-10-31

**Authors:** Ursula Neu, Zaigham Mahmood Khan, Benjamin Schuch, Angelina S. Palma, Yan Liu, Michael Pawlita, Ten Feizi, Thilo Stehle

**Affiliations:** 1 Interfaculty Institute of Biochemistry, University of Tuebingen, Tuebingen, Germany; 2 Glycosciences Laboratory, Department of Medicine, Imperial College London, London, United Kingdom; 3 Department of Genome Modificati and Carcinogenesis (F020), German Cancer Research Center, Heidelberg, Germany; 4 Department of Pediatrics, Vanderbilt University School of Medicine, Nashville, Tennessee, United States of America; University of Muenster – ZMBE, Germany

## Abstract

B-Lymphotropic Polyomavirus (LPyV) serves as a paradigm of virus receptor binding and tropism, and is the closest relative of the recently discovered Human Polyomavirus 9 (HPyV9). LPyV infection depends on sialic acid on host cells, but the molecular interactions underlying LPyV-receptor binding were unknown. We find by glycan array screening that LPyV specifically recognizes a linear carbohydrate motif that contains α2,3-linked sialic acid. High-resolution crystal structures of the LPyV capsid protein VP1 alone and in complex with the trisaccharide ligands 3′-sialyllactose and 3′-sialyl-*N*-acetyl-lactosamine (3SL and 3SLN, respectively) show essentially identical interactions. Most contacts are contributed by the sialic acid moiety, which is almost entirely buried in a narrow, preformed cleft at the outer surface of the capsid. The recessed nature of the binding site on VP1 and the nature of the observed glycan interactions differ from those of related polyomaviruses and most other sialic acid-binding viruses, which bind sialic acid in shallow, more exposed grooves. Despite their different modes for recognition, the sialic acid binding sites of LPyV and SV40 are half-conserved, hinting at an evolutionary strategy for diversification of binding sites. Our analysis provides a structural basis for the observed specificity of LPyV for linear glycan motifs terminating in α2,3-linked sialic acid, and links the different tropisms of known LPyV strains to the receptor binding site. It also serves as a useful template for understanding the ligand-binding properties and serological crossreactivity of HPyV9.

## Author Summary

Viruses must engage specific receptors on host cells in order to initiate infection. The type of receptor and its concentration on cells determine viral spread and tropism, but for many viruses, the receptor and the mode of recognition by the virus are not known. We have characterized the structural requirements for receptor binding of B-lymphotropic polyomavirus (LPyV). This virus was originally isolated from African Green Monkey lymph node cultures and attracted interest because of its narrow tropism for a human tumor cell line. LPyV is also the closest relative of the recently discovered Human Polyomavirus 9 (HPyV9). We screened the LPyV coat protein VP1 on an carbohydrate microarray and found that it specifically recognizes a linear sugar motif that terminates in α2,3-linked sialic acid. We then determined the structures LPyV VP1 bound to these carbohydrates. The protein has a preformed, deeply recessed binding site for sialic acid. The binding site differs in both architecture and mode of recognition from the binding sites of other viruses. LPyV only binds linear carbohydrates that are able to penetrate into the binding slot.

## Introduction

The B-Lymphotropic Polyomavirus (LPyV) was originally isolated from African Green Monkey lymph node cultures [1] and attracted interest because of its narrow tropism for the human B-lymphoblastoid tumor cell line BJA-B. In addition, significant antibody binding to LPyV was observed in human sera, raising the possibility that LPyV, or an LPyV-like human polyomavirus, might be a human oncovirus [2]. Polyomaviruses are a growing family of non-enveloped, icosahedral DNA viruses. Several members of the polyomavirus family, such as Simian Virus 40 (SV40), can transform cells in culture and cause tumors in animals [3], [4]. The recently discovered human Merkel Cell Polyomavirus (MCPyV) is clearly implicated in a human cancer [5]. However, infections by the human JC and BK Polyomaviruses (JCPyV and BKPyV, respectively) remain subclinical in healthy individuals and cause severe acute disease, not cancer, in immunocompromised patients [6]. It is not known whether LPyV is endemic in humans, but a closely related virus, Human Polyomavirus 9 (HPyV9), was identified in 2011 and detected in human serum, plasma, urine and skin [7], [8]. Although there are no data yet on the pathogenicity of HPyV9 in the human population, the seroprevalence is 47% in adults [9].

The narrow cell tropism of LPyV has made it a paradigm for studying viral tropism. Attachment of the LPyV major capsid protein VP1 to receptors on host cells is clearly critical for the restricted tropism of the virus [10]. Sialic acid is a crucial component of the LPyV receptor as removal or modification of cellular sialic acids abolished or modulated LPyV cell binding and infection [10], [11], [12], but the identity of the sialylated LPyV receptor and its role in tropism are not known. Sialic acids are a group of acidic monosaccharides that are based on neuraminic acid and decorate eukaryotic cell surfaces. The most prevalent sialic acid in humans is 5-*N*-acetyl-neuraminic acid (Neu5Ac) [13]. Sialic acids occur with different modifications and glycosidic linkages at the peripheral domains of a diversity of carbohydrate sequences of glycoproteins and glycolipids. Initial cell contacts by many viruses involve sialylated glycans [14]. In most cases, the interrelationship between the recognition of specific carbohydrate sequences by viruses and the effects on viral tropism and pathogenesis are only beginning to emerge.

Several polyomaviruses use specific sialylated carbohydrates as receptors [15], [16], [17]. Structural studies have shown that carbohydrate receptors are bound in shallow grooves on the polyomavirus capsid surface, which is formed by 72 pentamers of the major capsid protein VP1 [17], [18], [19], [20], [21].

In order to advance an understanding of the receptor-binding properties of LPyV, we expressed and purified its VP1 pentamers and subjected them to screening on a glycan microarray featuring a diverse set of sialylated carbohydrates. We detected specific and restricted binding only to the short trisaccharide probes, 3′-sialyllactose (3SL) and 3′-sialyl-*N*-acetyllactosamine (3SLN), and solved crystal structures of both glycans in complex with LPyV VP1. The structures reveal a preformed, recessed binding site for sialic acid that is different in architecture and location from known sialic acid binding sites of other viruses, and that essentially buries the sialic acid. Due to the high level of sequence similarity to HPyV9 VP1, we are also able to draw conclusions about the structure and receptor binding properties of this newly discovered human virus as well as its serological cross-reactivity with LPyV.

## Results

### LPyV VP1 specifically binds to a linear α2,3-sialylated carbohydrate motif

In order to elucidate the carbohydrate-binding specificity of LPyV in a controlled setting, we recombinantly expressed VP1 pentamers that are unable to assemble into capsids due to truncations of 27 and 66 residues at the N- and C-termini, respectively. These truncations do not affect the overall structure and receptor-binding properties of VP1 pentamers, as demonstrated by structure-function studies of related polyomavirus VP1 pentamers [17], [20], [21], [22] and their comparison with structures of entire virus particles [18], [23].

**Figure 1:**
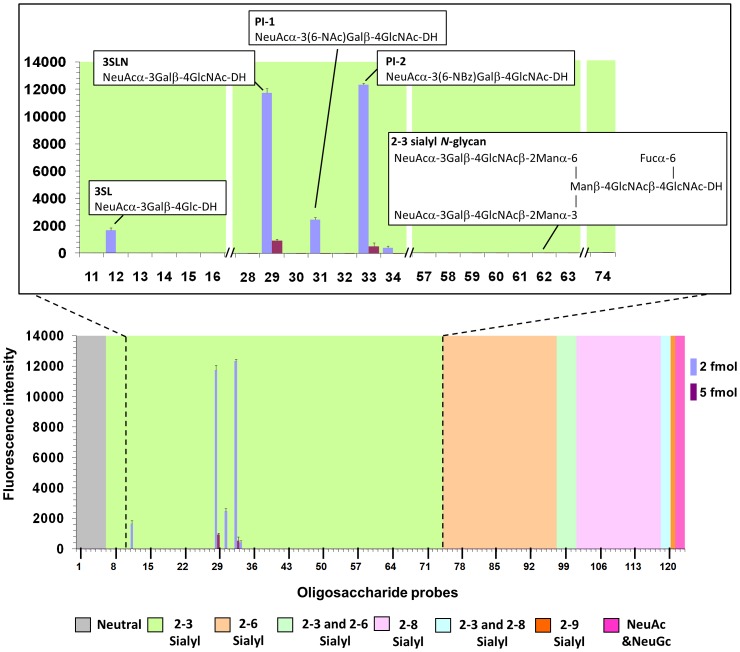
Glycan microarray screening analysis of LPyV VP1 pentamers. Glycan microarray analysis of LPyV VP1 showing selective binding to the short 3SL and 3SLN-related trisaccharide probes with ring-opened flexible cores but not to the *N*-glycan probe with 3SLN-terminating antennae, complex gangliosides, nor to any of the 6SL and 6SLN-related probes. Numerical scores for the binding intensity are shown as means of fluorescence intensities of duplicate spots at 2 and 5 fmol/spot. Error bars represent half of the difference between the two values. The microarrays consisted of lipid-linked oligosaccharide probes and the sequences are listed in Table S1. The probes are arranged according to terminal sialic acid linkage, oligosaccharide backbone chain length and sequence. The various types of terminal sialic acid linkage are indicated by the colored panels as defined at the bottom of the figure. The expanded region of the microarray highlights the sequences of the probes bound and the selective binding of LPyV VP1 to 3SL and 3SLN-related trisaccharide probes.

The purified pentamers were analyzed on a glycan microarray containing 117 sialylated and 6 non-sialylated lipid-linked oligosaccharide probes, representing sequences occurring on *N*- and *O*-linked glycoproteins as well as glycolipids. We detected LPyV VP1 binding signals above background to α2,3-sialylated probes bearing sequences related to 3SL and 3SLN (Fig. 1, probes 12 and 29). Both 3SL and 3SLN are linear trisaccharides with sequences Neu5Ac-α2,3-Gal-β1,4-Glc and Neu5Ac-α2,3-Gal-β1,4-GlcNAc, respectively (Fig. 1 and Table S1). The two additional probes (31 and 33) that yielded signals are chemically synthesized derivatives of 3SLN with additional 6-*N*-acetyl and 6-*N*-benzoyl functional groups, respectively, at the Gal moiety. There was no binding to a 3SL-derived structure bearing 4-*O*-acetylated Neu5Ac (probe 6), nor to the α2,6-linked sialyl analogs of 3SL and 3SLN (probes 77 and 81) or the α2,8-linked disialyl analog of 3SL (probe 104). A 3SLN analog with a β1,3 linkage between Gal and GlcNAc (probe 27) did not elicit a signal. In addition, ganglioside sequences that are branched at the Gal residue (e.g. probes 67 and 72) were not recognized. Taken together, glycan microarray analysis shows that binding to 3SL/3SLN is specific for defined linkages between each disaccharide, and only select modifications of individual sugar moieties can be tolerated.

There were three unusual observations in LPyV binding on the microarrays: First, there were no binding signals detected with longer oligosaccharide sequences with the same α2,3-sialyl trisaccharide terminus, such as probes with sialyl-lacto-*N*-neo-tetraose sequences (probes 42 and 43) and the sialyl-*N*-glycan (probe 62). Second, there was stronger binding to the ligand-positive probes at low levels (2 fmol per spot) compared to higher levels (5 fmol per spot) (Fig. 1). Third, strong LPyV binding was detected only to 3SL and 3SLN oligosaccharide probes that were linked to lipid by reductive amination and thus have ring-opened, reduced monosaccharide cores [24]. The probes with the same oligosaccharide sequences but prepared via oxime ligation without reduction [25] were not bound (probes 13 and 30), nor were glycosylceramides (probes 8–11). The reduced monosaccharide cores most likely increase the flexibility of the glycans, thereby facilitating engagement. We observed a related but less pronounced phenomenon with VP1 pentamers of SV40, which also elicited lower binding signals for receptor oligosaccharides on glycosylceramides than to the same sequences in the neoglycolipids prepared by reductive amination (Figure S1).

### Structure of LPyV VP1

**Figure 2:**
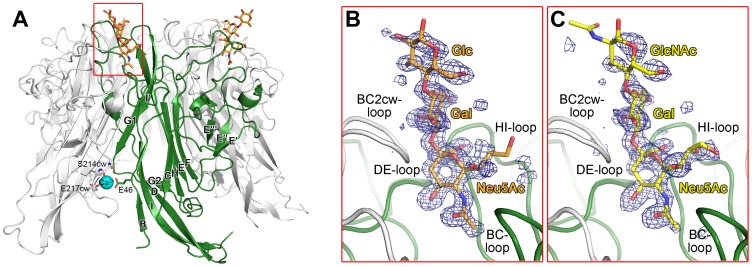
Structure an LPyV VP1 carbohydrate receptor complex. A. Overview of the LPyV VP1 structure in complex with 3SL. A LPyV VP1 pentamer is shown in cartoon representation, with one monomer highlighted in green. The oligosaccharide ligand is drawn in stick representation and colored by element, with carbons in orange, nitrogens in blue and oxygens in red. The calcium ion bound to the free LPyV VP1 pentamer is shown as a cyan sphere, with LPyV residues contacting it shown in stick representation. Residues 28–38 at the N-terminus are involved in non-native crystal contacts and are not shown here for clarity. B. and C. Close-up views of the 3SL (B) and 3SLN (C) binding sites. LPyV VP1 is drawn as in (A). The oligosaccharides are shown in stick representation, with 3SL in orange and 3SLN in yellow. Composite annealed omit difference density maps are shown contoured at 2.5 σ with a radius of 3.5 Å around the respective oligosaccharides.

In order to provide a structural basis for the observed interactions, we solved crystal structures of unliganded LPyV VP1 as well as complexes of LPyV VP1 with 3SL and 3SLN at resolutions of 1.92, 1.48 and 1.75 Å, respectively (Fig. 2, Table 1). All crystals contained two VP1 pentamers in their asymmetric units, and each model comprises 10 polypeptide chains. LPyV VP1 forms a ring-shaped homopentamer in which the five monomers are arranged around a central five-fold axis and connected by extensive interaction surfaces (Fig. 2A). At the core of each monomer, two antiparallel β-sheets, consisting of β-strands termed B, I, D, G and C, H, E, F, respectively, form a compact β-sandwich. To facilitate discussion of single residues in the symmetric, ring-shaped pentamer, one monomer will serve as reference monomer with no special designation; its clockwise and counterclockwise neighbors will be denoted cw and ccw, respectively.

**Table 1:** Data collection and refinement statistics.

	LPyV VP1 Native	LPyV VP1+3SL	LPyV VP1+3SLN
**Data Collection**			
Space Group	C2	C2	C2
Unit cell dimensions:			
a, b, c (Å)	150.0, 95.9, 231.7	150.5, 97.2, 234.7	150.2, 97.2, 234.2
α, β, γ (°)	90, 95.8, 90	90, 96.2, 90	90, 96.2, 90
Resolution (Å)^a^	40 – 1.92 (1.97 – 1.92)	50 – 1.48 (1.52 – 1.48)	50 – 1.75 (1.80 – 1.75)
Total reflections	730,974 (55,734)	1,654,072 (121,178)	1,030,657 (76,191)
Unique Reflections	246,146 (18,268)	549,960 (40,660)	333,435 (24,336)
<I>/<σ(I)>	10.5 (1.7)	17.5 (2.4)	14.6 (2.3)
Completeness (%)	98.8 (99.5)	98.6 (98.5)	99.0 (98.1)
R_meas_ (%)	9.4 (75.3)	4.2 (60.7)	6.5 (59.3)
Wilson B-factor (Å^2^)	32.3	25.0	27.3
**Refinement**			
R_work_ (%)	16.5 (25.7)	16.5 (24.0)	16.5 (24.7)
R_free_ (%)^b^	19.8 (29.0)	18.5 (25.1)	19.5 (27.9)
Coordinate error (Å)^c^	0.20	0.17	0.20
No. of atoms:			
Protein	21,149	21,395	21,354
Oligosaccharide	–	310	238
Water	1,877	2,828	2,900
Others^d^	118	188	152
Average B-factors (Å^2^):			
Protein	28.7	21.4	25.1
Oligosaccharide	–	33.8	42.4
Water	35.7	30.1	33.0
Others^d^	42.4	34.4	34.8
RMSD bond length (Å)	0.008	0.008	0.009
RMSD bond angle (°)	1.3	1.3	1.3
Ramachandran plots^e^			
Most favorable (%)	97.80	97.72	97.72
Allowed (%)	2.13	2.38	2.38
Outlier (%)	0.07	0	0

a) The highest resolution shell is shown in parenthesis. A single crystal was used for each data set.

b) 5% of total reflections were used to calculate R_free_.

c) Coordinate error (Luzzati plot) was calculated using sfcheck program of the CCP4 suite.

d) Compounds from the crystallization solution (isopropanol, calcium, chloride & ethylene glycol).

e) Calculated using the Molprobity server (http://molprobity.biochem.duke.edu/).

The LPyV VP1 structure contains one bound Ca^2+^ ion per chain (Fig. 2A) that is coordinated by the carboxylate group of E46 and the carbonyl group of S214cw as well as several water molecules. In addition, the carboxylate group of E217cw is close enough to the Ca^2+^ to engage in an ionic interaction. These residues are highly conserved among all polyomaviruses and form one of the two calcium binding sites in the SV40 virion that are important for capsid stability and regulating assembly [23]. In the virion, the Ca^2+^ coordination is completed by a glutamate residue of the incoming C-terminal arm from another VP1 pentamer. While the Ca^2+^ ions in our structures likely come from the crystallization solution containing 0.2 M calcium chloride, they nevertheless demonstrate that unassembled VP1 pentamers can weakly bind Ca^2+^ ions.

### Structures of LPyV VP1 - oligosaccharide complexes

We observed clear electron density for the oligosaccharide ligands in crystals soaked in 3SL and 3SLN. In contrast, crystals soaked in the same concentration of 6′-sialyllactose (6SL) did not yield any electron density for the glycan (data not shown), confirming the specificity found by glycan array screening. Each LPyV VP1 pentamer contains five oligosaccharide binding sites, which are located at the top of the pentamer (Fig. 2A–C), corresponding to the outer surface of the virion. Only some of these binding sites were occupied with ligand, whereas access to the remaining binding sites was blocked by crystal contacts. Both compounds bound in essentially the same manner to each binding site.

**Figure 3:**
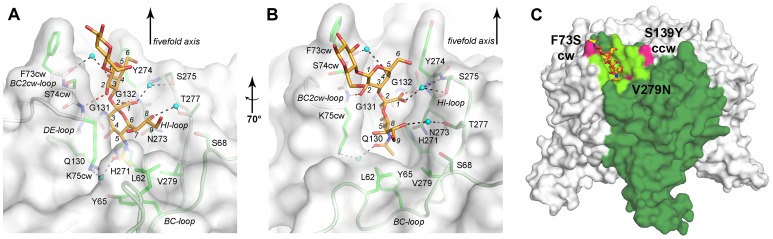
Specific interactions of LPyV VP1 and 3SL. A and B. LPyV VP1 is shown in cartoon and surface representation, with side chains interacting with 3SL shown in stick representation. The 3SL oligosaccharide is shown in stick representation and colored by element, with carbons in orange, nitrogens in blue and oxygens in red. Waters are represented with spheres. Selected carbon atoms of 3SL are numbered in italics. Residues engaging in hydrophobic interactions with 3SL are colored bright green and residues forming polar van der Waals contacts or water-mediated hydrogen bonds are colored dark green. Direct hydrogen bonds between LPyV VP1 and the oligosaccharide are shown as red dashed lines, water-mediated hydrogen bonds are colored black. The views depicted in A and B differ by a 70° rotation around a vertical axis. C. Location of LPyV VP1 mutations influencing cell tropism. An entire LPyV VP1 pentamer is shown in surface representation. The N-terminal arms were omitted for clarity. One monomer is colored dark green, and the residues forming the oligosaccharide binding site are highlighted in light green. Residues whose mutation was shown to influence cell tropism of LPyV VP1 are colored magenta. The 3SL and 3SLN oligosaccharides are shown in stick representation and colored orange and yellow, respectively.

The linear 3SL and 3SLN chains can assume a range of possible conformations in solution due to rotational freedom of the glycosidic bonds [26], [27]. LPyV VP1 binds both compounds in a conformation of the Neu5Ac-α2,3-Gal linkage that is preferred in solution (mean torsion angles 65°, −26°) [26], [27]. The terminal Neu5Ac residue, which is best defined by electron density and has low temperature factors (B-factors), inserts deeply into a cleft and engages the protein with multiple contacts (Fig. 3A,B). The adjacent Gal makes fewer contacts and has elevated B-factors. The terminal Glc and GlcNAc residues of 3SL and 3SLN have the weakest electron density and are only visible in the best occupied sites in each structure (Fig. 2B,C).

### Interactions between VP1 and carbohydrate residues

Each carbohydrate binding site lies at the contact between two VP1 monomers and is formed by residues from the BC-, DE- and HI-loops of one monomer as well as the BCcw-loop of the clockwise neighboring monomer (Fig. 2B,C). The long BC-loop can be divided into two substructures, termed BC1- and BC2-loop, that point into different directions. In contrast to most viral sialic acid-binding sites [14], the LPyV site is not a shallow depression on the protein surface, but a deep cleft that contacts both faces of the Neu5Ac ring (Fig. 3A,B), mostly *via* van der Waals interactions. Consequently, 80% of the accessible surface area of Neu5Ac (317 Å^2^ of a total of 399 Å^2^) is buried upon binding. The methyl group projects most deeply into the binding site and is surrounded by the side chains of L62, Y65, Q130, H271 and V279 on three different VP1 loops (Fig. 3A). These interactions bury the entire accessible surface (72 Å^2^) of the methyl group. In addition, there is a water-mediated hydrogen bond between the Neu5Ac carbonyl group and K75cw on the BC2cw-loop. The Neu5Ac binding pocket is characterized by a high level of surface complementarity for its ligand (Fig. 3B). One face of Neu5Ac packs against the HI-loop of LPyV VP1 and makes van der Waals contacts with H271 and N273. On the same face, the carboxylate group of Neu5Ac is recognized by a hydrogen bond to the backbone amine of Y274 and water-mediated hydrogen bonds to the backbone amine and side chain of S275. The other face of Neu5Ac is covered with the hydrophobic part of the K75cw side chain in the BC2cw-loop, forming a lid that lies on top of the partially hydrophobic surface of the sugar ring. In addition to these interactions, residues Q130-G132 contact Neu5Ac from the rear of the binding site. The glycerol chain of Neu5Ac points away from the binding pocket, and its terminus adopts different conformations in different binding sites. However, the glycerol group engages in van der Waals contacts, a water-mediated hydrogen bond from O8 to T277 (Fig. 3B) and sometimes a water-mediated hydrogen bond from O9 to S68.

The Gal residue engages in fewer contacts that contribute about 25% of the total buried surface area. Contacts include a hydrogen bond between O2 of Gal and the backbone of S74cw and a water-mediated hydrogen bond between O4 of Gal and the backbone of F73cw, both in the BC2cw-loop. Moreover, there are van der Waals interactions with the Y274 side chain in the HI-loop (Fig. 2A). The terminal Glc and GlcNAc residues are only observed in few binding sites in both complexes. They are within 5 Å of the side chains of F73cw and S74cw. Although the hydrophobic, solvent-exposed side chain of F73cw is not well defined by electron density, it lies close enough to the Glc or GlcNAc residues to allow for weak van der Waals interactions. It is not clear from the structure whether these strengthen binding or cause weak steric hindrance.

In order to determine whether conformational changes occur in the protein during ligand binding, we compared the complex structures with the unliganded LPyV VP1 structure. In all chains in which the tip of the BC2cw-loop and adjacent residues are not engaged in crystal contacts, the structures are very similar, indicating that the binding site does not undergo a permanent induced fit movement. In two chains, however, a crystal contact perturbed the native structure of the BC2cw-loop, but not the ligand-bound one. Thus, complex formation might stabilize the receptor binding site, and there might be some flexibility in the unbound structure that allows ligand entry into the site. This hypothesis is supported by the B-factors of receptor-binding residues, which are elevated in the unbound structure compared with the complexes.

### Structural basis of specificity

The LPyV VP1 structures help to rationalize the specific recognition of the trisaccharide motif Neu5Ac-α2,3-Gal-β1,4-Glc(NAc) observed in glycan array screening. This motif can tolerate benzoyl or acetyl substituents at position 6 of the Gal ring, both of which are unlikely to interfere with binding (Fig. 3B). Whereas Neu5Ac in the context of 3SL was readily bound, the analogous α2,6-linked oligosaccharide 6SL was not recognized in both glycan array and crystal soaking experiments. Due to the α2,6-linkage, the overall shape of the trisaccharide is somewhat kinked and differs from the linear orientation found in α2,3-linked compounds. An α2,6-linkage would likely lead to loss of the hydrogen bonds involving Gal and clashes with the BC2cw- or HI-loops of LPyV VP1. Moreover, our structures explain the inability of LPyV VP1 to bind to an α2,8-linked disialic acid sequence. The Neu5Ac binding site can only accommodate terminal Neu5Ac, and a second Neu5Ac in a disialic acid sequence would occupy the place of the Gal residue, where it would cause steric clashes. Consistent with the results from glycan array screening, the β1,3 analog of 3SL would lead to clashes with protein residues in some conformations. Finally, LPyV VP1 does not bind to branched sequences that carry additional sugar residues attached to the Gal residue at position 4, such as gangliosides GM1 or GD1a. In both conformations of the α2,3-linkage that have been observed in such compounds, steric clashes with the protein would occur if residues were added at that position.

### Strain variations in receptor binding site

There are currently three different sequences of LPyV available in GenBank, which correspond to the K38, L02 and LPV-76 strains, which are all based on the same isolate (GenBank NC004763.1 (used here), AAA47067.1, AAA47076.1, respectively) [28], [29], [30]. The tropism of the K38 and L02 strains is restricted to proliferating B-lymphocyte lines, while the LPV-76 strain is also able to infect select T-lymphocyte lines [30]. The changes in tropism have been mapped to the VP1 proteins. The K38 and L02 VP1 proteins differ by three point mutations, while LPV-76 VP1 has three additional point mutations. Interestingly, the critical three amino acids linked to changes in tropism (F73S, S139Y and V/S279N) all cluster at the carbohydrate binding site (Fig. 3C). Our structure shows that none of the mutations would directly block binding of 3SL or 3SLN. The V/S279N mutation would only influence interactions with the terminal sialic acid, maybe altering specificity for modified sialic acids. The hydrophobic, but entirely solvent accessible side chain of F73 (Fig. 3C) can adopt two different conformations in the native structure, one of which approaches the Glc(NAc) residue in the oligosaccharide complexes. Mutation to serine could take away this weak contact or relieve steric hindrance and allow binding of a different carbohydrate. Similarly, S139Y might interfere with the binding of longer or branched oligosaccharides. While this residue is not part of the primary sialic acid binding site, it lies directly adjacent to it (Fig. 3C). Thus, our data would suggest that the different tropisms of the three strains are linked to small differences in receptor binding properties. Sialic acid is used by many viral attachment proteins as a tightly bound “hook” to grasp the oligosaccharide, while residues outside the sialic acid binding site modulate binding specificity for sialic acid in different contexts [14], [17], [31].

### Implications for HPyV9 structure

**Figure 4:**
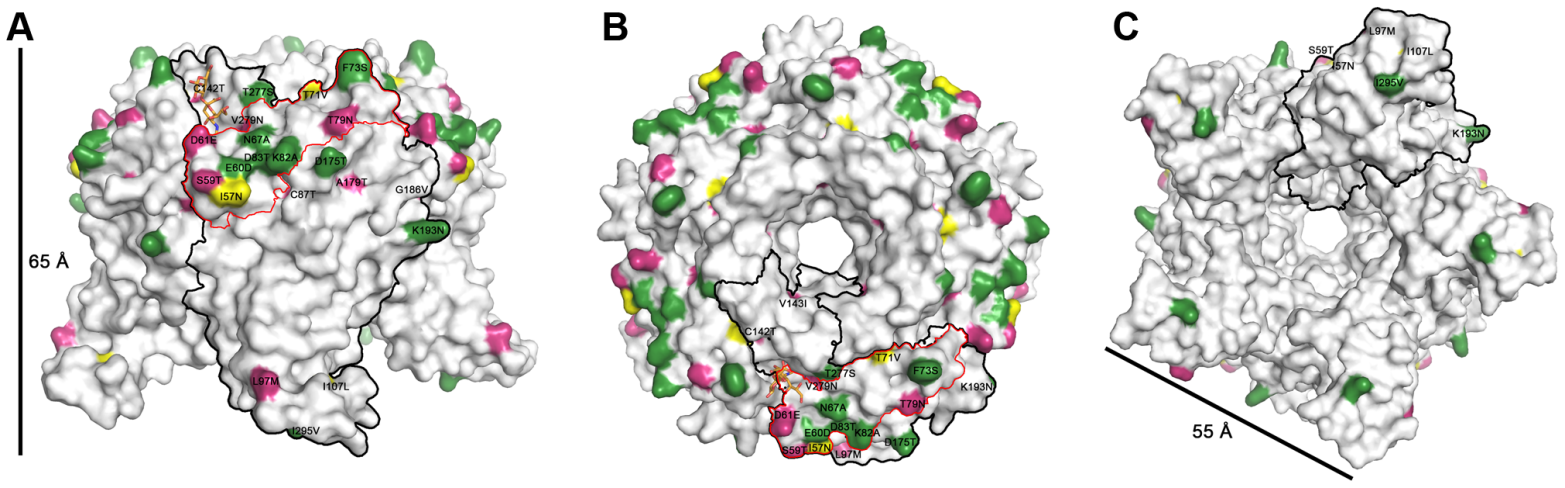
Mapping of differences between LPyV and HPyV9 VP1. The LPyV VP1 structure is shown in surface representation viewed from the side of VP1 (A), from the top of VP1 (B) and from the bottom of VP1 (C). The N-terminal arms were omitted for clarity. Residues that are unchanged between LPyV VP1 and HPyV9 are colored white, residues that are larger in LPyV VP1 than in HPyV9 are colored green, those larger in HPyV9 VP1 are colored magenta and those that are changed to a residue of equal size are colored yellow. The black outline encircles an LPyV VP1 monomer while the red outline delineates its BC-loop. Scale bars indicate the approximate dimensions of the pentamer.

LPyV is the closest homolog of the recently discovered human polyomavirus HPyV9 [7], [8]. The two VP1 proteins share 87% sequence identity, indicative of a high level of conservation at the structural level. HPyV9 had long been suspected to exist in the human population based on serological reactivity of human sera against LPyV [2], [32], and there is significant cross-reactivity for LPyV and HPyV9 in both human and African Green Monkey sera [9]. Surface-exposed LPyV VP1 residues show the same high level of sequence identity with HPyV9 VP1 as residues in the protein interior (87%). However, divergent residues are distributed unevenly on the LPyV VP1 surface, with most changes occurring on the top surface of VP1, which would be most accessible to antibodies in the context of the virion (Fig. 4). The long BC-loop (residues 55–85), which contributes most to this surface, is especially divergent (red line in Fig. 4). Surface residues of the BC-loop are only 52% identical with HPyV9 VP1 residues, while BC-loop residues facing towards the interior are 100% identical in sequence. The inner surface of the ring-shaped pentamer (Fig. 4C), which contacts the minor capsid proteins, and its side surface (Fig. 4A), which forms contacts between VP1 pentamers during capsid assembly, are entirely conserved between LPyV and HPyV9.

Most residues in the LPyV VP1 sialic acid binding site are conserved in HPyV9 VP1 (Fig. 4), and none of the substitutions would sterically interfere with Neu5Ac binding. This suggests that HPyV9 is also capable of interacting with sialylated oligosaccharides, and that the two viruses might even share a similar sialic acid binding mode. Interestingly, two of the tropism-widening substitutions among different LPyV strains are also present in HPyV9. However, the prediction of carbohydrate binding sites based on homology modeling alone remains challenging, and further studies are necessary to confirm the attachment of HPyV9 to sialylated oligosaccharides on host cells.

### Comparison with other polyomavirus binding sites

The interaction of LPyV VP1 with Neu5Ac in a narrow, slot-like binding site is unique among polyomavirus-receptor complexes [17], [20], [21], [22], and also among the many other viruses that engage sialic acids [14]. There is only one direct hydrogen bond between LPyV VP1 and Neu5Ac, whereas there are typically at least four and up to seven direct hydrogen bonds in other virus-sialic acid complexes [14]. To achieve specificity for Neu5Ac, the LPyV binding site seems instead to rely more on shape complementarity and van der Waals contacts than on a distinct pattern of hydrogen bonds.

**Figure 5:**
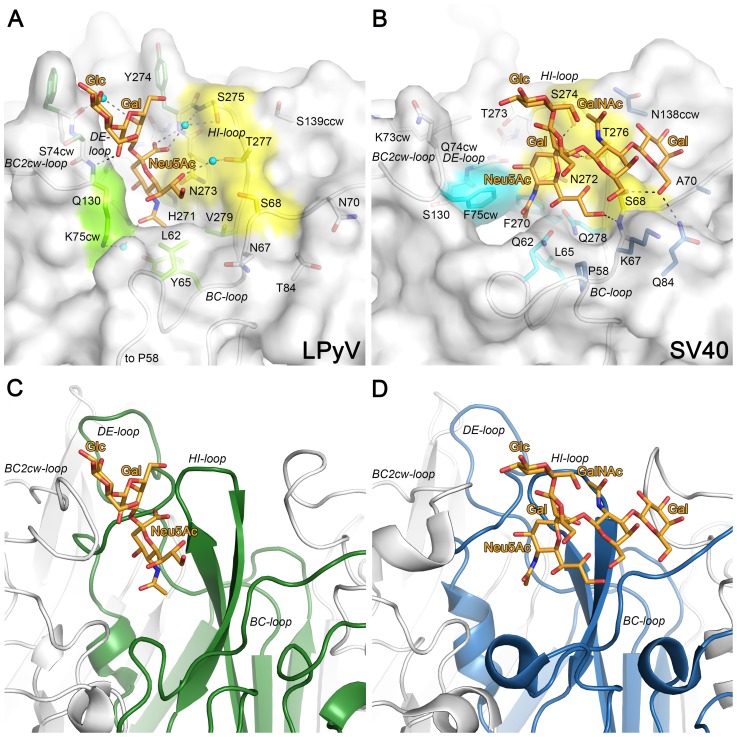
Comparison of oligosaccharide binding sites of LPyV and SV40. A & C. LPyV VP1 in complex with 3SL. B & D. SV40 VP1 in complex with GM1 pdb (3BWR). In panels A and B, the proteins are shown in surface representation, with the BC- and HI-loops also indicated in cartoon representation. Residues contributing to ligand binding or specificity are shown in stick representation. Receptor-binding residues that are identical between the two proteins are colored yellow in both panels, while residues that differ between the two proteins, but reside on the same location on the VP1 surface, are colored bright green for LPyV and cyan for SV40. Residues that make additional contacts with the oligosaccharide only in one complex are colored dark green for LPyV and dark blue for SV40. Their non-binding counterparts in the other complex are colored white. The carbohydrate ligands are shown as orange sticks. Hydrogen bonds and water-mediated hydrogen bonds are shown as black and grey dashes, respectively. In panels C and D, the proteins are shown in ribbon presentation, with one monomer highlighted in green for LPyV and blue for SV40 VP1, respectively.

Despite these differences, the LPyV binding site lies in a region that partially overlaps with the sialic acid binding sites on other polyomaviruses. LPyV engages Neu5Ac in an orientation that resembles that seen in the complex of SV40 VP1 with GM1, and it is therefore useful to compare the two modes of interaction (Fig. 5). Interestingly, the two binding sites are “half-conserved”. The HI-loop, which contributes the “back” wall of the sialic acid binding site, is conserved, while the BC1-, BC2cw- and DE-loops feature marked differences, which explain the different orientations of bound Neu5Ac. In the SV40 VP1 complex, the side chain of F75cw is a central hydrophobic contact for the Neu5Ac methyl group. This residue would interfere with the binding of Neu5Ac in the orientation observed in the LPyV VP1 complex. In LPyV VP1, replacement of F75cw with lysine, a different conformation of the BC2cw-loop and a more distant DE-loop create a recessed surface with an especially deep and narrow pocket that can accommodate Neu5Ac. As a consequence of these changes, the conserved residues of the HI-loop engage in different contacts with the two Neu5Ac orientations (Fig. 5). Taken together, the comparison highlights how the architecture of the sialic acid binding site, constructed from several loops, can be varied by mutation of some modules while conserving others. With LPyV, there are four known orientations of sialic acid in polyomavirus binding sites. Except for the LPyV-SV40 pair, none of the amino acids that contact them are conserved, but they tend to occupy equivalent positions in sequence and in structure. The observed partial conservation might be a general strategy for evolving binding sites with new properties through functional intermediates that minimize the risk of losing binding altogether.

## Discussion

In this study, we have established the linear, short sequence Neu5Ac-α2,3-Gal-β1,4-Glc(NAc) as a binding motif for the LPyV attachment protein VP1, solved X-ray structures of LPyV VP1 in complex with two cognate oligosaccharide ligands, and defined contacts in the sialic acid binding site.

Neu5Ac clearly serves as the primary point of contact for LPyV, in agreement with previous data showing that LPyV binding and infection were dramatically decreased upon neuraminidase treatment of cells [10], [33]. Cells treated with modified sialic acid precursors and thus bearing modified sialic acids were found to no longer support LPyV infection if the sialic acids contained long acyl chains on the *N*-substituent [11]. These longer chains would clash with the side chains of Y65 or V279 in the sialic acid binding site (Fig. 3B). Cells that had incorporated 9-iodo-Neu5Ac and 5-*N*-fluoroacetyl-Neu5Ac exhibited increased LPyV infection compared with cells carrying unmodified Neu5Ac [12]. In 9-iodo-Neu5Ac, the outermost hydroxyl group of the glycerol chain is replaced with the bigger and more hydrophobic iodine. The iodine could interact favorably with a hydrophobic patch on the LPyV VP1 surface formed mainly by the L62 side chain (Fig. 3B). This patch might also conceivably interact with parts of the naturally occuring 9-*O*-acetyl Neu5Ac, which was not present in our arrays in the context of 3SL or 3SLN. 5-*N*-fluoroacetyl-Neu5Ac carries a polar fluorine attached to the *N*-acetyl group, which could likely be accommodated by the binding site defined in our structure.

The natural host of LPyV is the African Green Monkey, in which the predominant sialic acid, 5-*N*-glycolyl neuraminic acid (Neu5Gc) carries an additional hydroxyl group attached to the *N*-acetyl group. Like 5-*N*-fluoroacetyl-Neu5Ac, Neu5Gc could also be accommodated by LPyV VP1, reflecting the host preference of the virus.

Glycan array and structural analyses show that LPyV VP1 specifically recognizes linear trisaccharides terminating in α2,3-linked sialic acid. It was previously shown that LPyV and SV40, which binds the branched α2,3-sialylated glycolipid GM1, do not compete for receptors on host cells [10]. This finding can easily be rationalized because the branched GM1 sequence would clash with LPyV VP1 residues. Interestingly, the short binding sequence we describe here contrasts with the longer glycan sequence required by JCPyV [17], which may be quite restricted in cellular expression. The 3SL/3SLN sequence on the other hand is present on glycoproteins and glycolipids of many cell types. Why then is there a narrow cell tropism of LPyV in human cell lines? The trisaccharide 3SL corresponds to the glycan portion of the ganglioside GM3, a possible receptor candidate. However, the Glc residue in GM3 would most likely be buried in the head group layer of the membrane [34], and modelling suggests that membrane-bound GM3 would clash with LPyV residue F73 when the Neu5Ac is inserted into the binding pocket. It is however possible that GM3 can be engaged in certain contexts, for example when linked to a specific membrane anchor [35]. The 3SL/3SLN trisaccharide sequence could also be part of a longer, yet uncharacterized oligosaccharide chain. Further work is required to determine whether the receptor for LPyV is a glycolipid with a particular ceramide moiety as discussed in [36] or possibly a glycoprotein that displays a particular cluster of sialyl trisaccharides and that perhaps also contributes to tropism.

Based on our microarray data, the narrow tropism could also be, at least in part, due to a requirement for a particular mode of the presentation of the short sialyl motif to elicit binding of the VP1 pentamer, similar to what has been observed in other cases [36]. On the array, the glycans have flexible linkages to lipid, and the non-covalent attachment of the probes to the nitrocellulose matrix in the presence of carrier lipids [37] provides them with an element of mobility that enables them to be presented in the required geometry as long as they are not too densely packed. Assuming that the glycan probes are distributed uniformly over the surface of a spot, the distances between one glycan and a neighbouring glycan would be 20–30 Å at 5 fmol/spot, and 40–50 Å at 2 fmol/spot. Interestingly, these distances are in the same range as the distances between two binding sites on a pentamer, which are 30 Å for adjacent and 47 Å for non-adjacent sites. It is therefore at least conceivable that a 5 fmol/spot concentration of glycans does not allow for an effective interaction of more than one glycan with the LPV pentamer, perhaps for steric or entropic reasons. The unusual ligand-binding site of LPyV may therefore reflect a strategy to efficiently engage less densely packed, more accessible ligands at the cell surface.

## Materials and Methods

### Protein expression and purification

DNA coding for amino acids 28–301 of LPyV VP1 (K38 strain, GenBank accession no. NC 004763.1) was amplified by PCR and cloned into the pET15b expression vector (Novagen) in frame with an N-terminal hexahistidine tag (His-tag) and a thrombin cleavage site. The protein was overexpressed in *E. coli* BL21(DE3) and purified by nickel affinity chromatography. For glycan array screening and crystallization, the protein was further purified by gel filtration on Superdex-200. For crystallization, the His-tag was cleaved with thrombin before the gel filtration step, leaving the non-native amino acids GSHM at the N-terminus. After gel filtration, the protein was kept in a buffer comprised of 20 mM HEPES pH 7.5, 150 mM NaCl and 10 mM DTT.

### Glycan array screening

The microarray was composed of 123 sequence-defined lipid-linked oligosaccharide probes: 117 sialyl-terminating probes and 6 neutral probes as negative controls (Glycosciences Array Set 30–31, Table S1). The probes were robotically printed in duplicate on nitrocellulose-coated glass slides at 2 and 5 fmol per spot using a non-contact instrument [38], [39]. The his-tagged recombinant VP1 protein was pre-complexed with mouse monoclonal anti-poly-histidine (Ab1) and biotinylated anti-mouse IgG antibodies (Ab2) (both from Sigma) in a ratio of 4∶2∶1 (by weight). In brief, the LPyV VP1-His tagged protein-antibody pre-complexes were prepared by pre-incubating Ab1 with Ab2 for 15 min at ambient temperature, followed by addition of VP1 and incubation for an additional 15 min on ice. The VP1-antibody complexes were diluted in 5 mM HEPES pH 7.4, 150 mM NaCl, 3% (w/v) bovine serum albumin (Sigma) and 5 mM CaCl_2_, to give a final VP1 concentration of 150 µg/ml in the presence of 2.2 mM DTT, and overlaid onto the arrays at 20°C for 2 h. Binding was detected with Alexa Fluor-647-labelled streptavidin (Molecular Probes); imaging and data analysis was as described [38], [40].

### Crystallization and structure determination

LPyV VP1 was crystallized by sitting drop vapor diffusion at a concentration of 7 mg/mL against a reservoir comprising 22% (v/v) isopropanol, 0.1 M sodium acetate pH 4.8 and 0.2 M calcium chloride. For complex formation, LPyV VP1 was crystallized by hanging drop vapor diffusion and microseeding at a lower protein concentration of 3.8–4.25 mg/mL against a reservoir that containing a lower isopropanol concentration of 12% (v/v). Crystals were harvested into the respective reservoir solutions and cryoprotected by soaking them for 10 s in reservoir solution containing 25% (v/v) ethylene glycol. They were then flash-frozen in liquid nitrogen. For complex formation, crystals were soaked in reservoir solution supplemented with 40 mM 3′-sialyllactose (Dextra, UK) or 40 mM 3′-sialyllactosamine (Carbosynth, UK) for 45 and 10 min, respectively. They were then cryoprotected in reservoir solution supplemented with 25% (v/v) ethylene glycol and 40 mM oligosaccharide, and flash-frozen.

Diffraction data were collected at ESRF (Grenoble, F) (beamline ID23-1) and at SLS (Villigen, CH) (beamlines X06SA and X06DA). Data were processed with xds [41], and the structure was solved by molecular replacement with Phaser in CCP4 [42], [43] using the β-sandwich core of the mPyV VP1 pentamer structure (1VPS) as a search model [22]. The crystals belong to space group C2 with two pentamers in their asymmetric unit (Table 1). After rigid body and simulated annealing coordinate refinement in Phenix [44], missing parts of the model such as the surface loops appeared in electron density maps and could be built in Coot [45]. Refinement proceeded by alternating rounds of restrained coordinate, isotropic B-factor and TLS refinement in Phenix or Refmac5 [46], and model building in Coot. The non-crystallographic symmetry relating the ten LPyV VP1 monomers in the asymmetric unit was used as a restraint throughout refinement. In data from soaked crystals, the ligands were located in weighted 2 mF_o_-DF_c_ and mF_o_-DF_c_ electron density maps. The carbohydrates were refined using restraints from the CCP4 library, with the exception of the α2,3-glycosidic bond, which had to be user-defined. Waters were incorporated using Coot and ARP/wARP. The final models have good stereochemistry and low R_free_ values [47] (Table 1). Residues 28–98 and 106–297 could be modeled for all 10 chains in all structures, with one loop being disordered and additional residues at the C-termini visible in a subset of chains. In addition, the vector-encoded sequence SHM was observed at the N-terminus in all copies. Coordinates and structure factor amplitudes were deposited with the RCSB data bank (www.rcsb.org) with entry codes 4MBX (unliganded LPyV VP1), 4MBY (complex with 3SL) and 4MBZ (complex with 3SLN). Figures showing the X-ray structures were prepared with PyMol (Schrödinger Inc.).

## Supporting Information

Figure S1 Glycan microarray analysis of SV40 VP1 showing selective binding to GM1-type ganglioside probes. Numerical scores for the binding intensity are shown as means of fluorescence intensities of duplicate spots at 2 and 5 fmol/spot. Error bars represent half of the difference between the two values. The microarrays consisted of lipid-linked oligosaccharide probes and the sequences are listed in Table S1. The probes are arranged according to terminal sialic acid linkage, oligosaccharide backbone chain length and sequence. The various types of terminal sialic acid linkage are indicated by the colored panels as defined at the bottom of the figure. The inset highlights the selective binding of SV40 VP1 to GM1 probes carrying either N-acetyl-neuraminic acid (GM1Ac) or N-glycolyl neuraminic acid (GM1Gc) immobilized with glycolipid (GL) or neoglycolipid (NGL) linkers.
(JPG)

Table S1 This table lists oligosaccharide probes included in the Glycosciences Array Set 30–31, sorted by sialyl linkage and backbone-type sequences, and the binding signals (fluorescence intensities at 2 fmol/probe) they elicited with LPyV VP1.
(DOC)
