# Supplementary figures and images for: Structures of B-Lymphotropic Polyomavirus VP1 in Complex with Oligosaccharide Ligands
Source: PLoS Pathog. 2013 Oct 31;9(10):e1003714. doi: 10.1371/journal.ppat.1003714 (PMC3814675; doi:10.1371/journal.ppat.1003714)

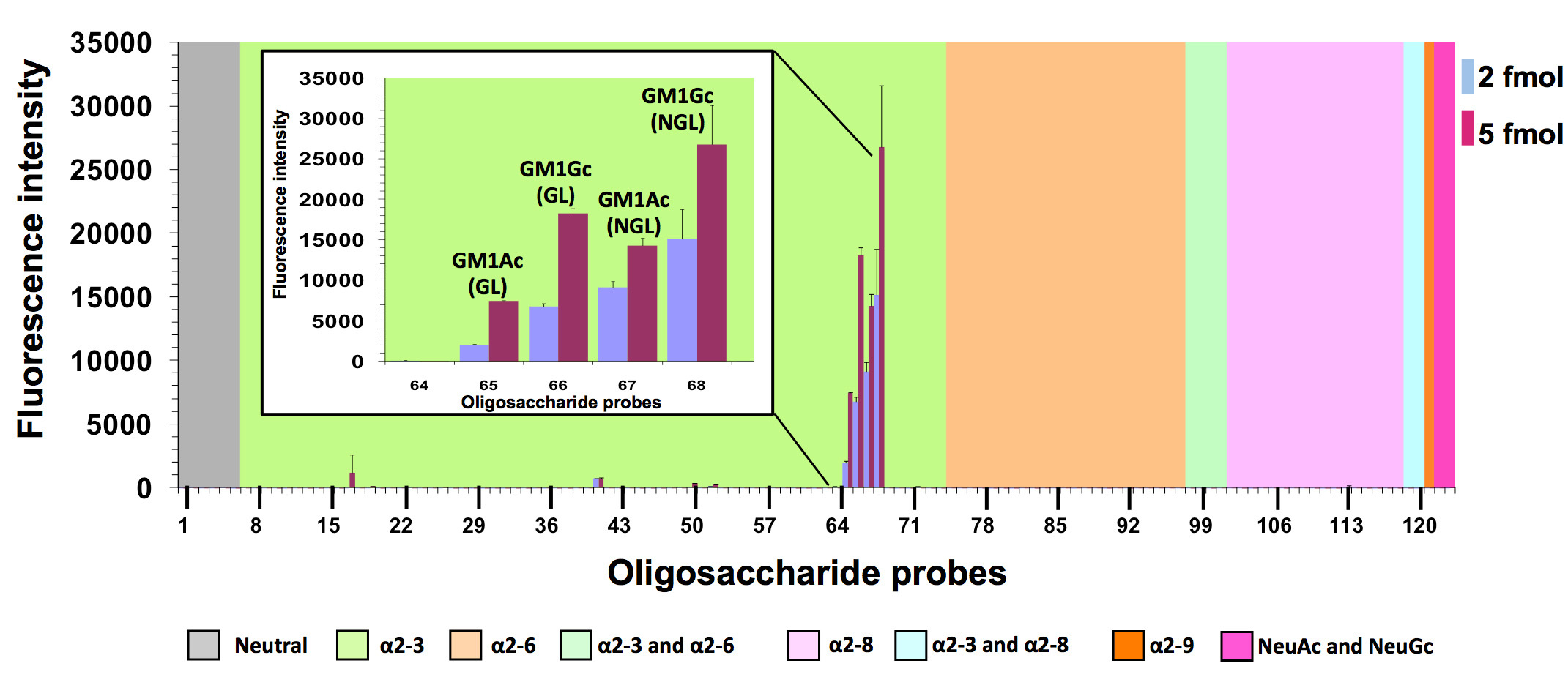

Supplement: Figure S1 — Glycan microarray analysis of SV40 VP1 showing selective binding to GM1-type ganglioside probes. Numerical scores for the binding intensity are shown as means of fluorescence intensities of duplicate spots at 2 and 5 fmol/spot. Error bars represent half of the difference between the two values. The microarrays consisted of lipid-linked oligosaccharide probes and the sequences are listed in Table S1. The probes are arranged according to terminal sialic acid linkage, oligosaccharide backbone chain length and sequence. The various types of terminal sialic acid linkage are indicated by the colored panels as defined at the bottom of the figure. The inset highlights the selective binding of SV40 VP1 to GM1 probes carrying either N-acetyl-neuraminic acid (GM1Ac) or N-glycolyl neuraminic acid (GM1Gc) immobilized with glycolipid (GL) or neoglycolipid (NGL) linkers. (JPG) [file ppat.1003714.s001.jpg]
