# Supplementary material for: Structures of B-Lymphotropic Polyomavirus VP1 in Complex with Oligosaccharide Ligands
Source: PLoS Pathog. 2013 Oct 31;9(10):e1003714. doi: 10.1371/journal.ppat.1003714 (PMC3814675; doi:10.1371/journal.ppat.1003714)
Supplement: Table S1 — This table lists oligosaccharide probes included in the Glycosciences Array Set 30–31, sorted by sialyl linkage and backbone-type sequences, and the binding signals (fluorescence intensities at 2 fmol/probe) they elicited with LPyV VP1. (DOC) [file ppat.1003714.s002.doc]

**Supporting Table S1:** Oligosaccharide probes^a^ included in the Glycosciences Array Set 30-31, sorted by sialyl linkage and backbone-type sequences, and the binding signals (fluorescence intensities at 2 fmol/probe) they elicited with LPyV VP1.

| **ID** | ***Sequence*** | **Name** | **Fluorescence signal intensities**^b^ |
| --- | --- | --- | --- |
|  |  |  | **LPyV-VP1** |
|  | ***Neutral*** |  |  |
| 1 | Galß-4GlcNAc-AO | LacNAc | - |
| 2 | Galß-3GlcNAcß-3Galß-4Glc-DH | LNT | - |
| 3 | Galß-4GlcNAcß-3Galß-4Glc-DH | LNnT | - |
| 4 | Galß-4GlcNAcß-3Galß-4Glc-DH  │  Fucα-3 | LNFP-III | - |
| 5 | Galß-4GlcNAcß-6  │  Galß-4Glc-DH  │ Galß-3GlcNAcß-3 | LNH | - |
| 6 | Galß-4GlcNAcß-2Manα-6   │   Manß-4GlcNAcß-4GlcNAc-DH  │  Galß-4GlcNAcß-2Manα-3 | NA2 | - |
|  | ***2-3 Sialyl***  ***Gal, Lactose and N-acetyllactosamine-based linear backbones*** |  |  |
| 7 | NeuAcα-3Galβ-Cer | GM4 | - |
| 8 | NeuAcα-3Galß-4Glcß-Cer | GM3 | - |
| 9 | NeuGcα-3Galß-4Glc-Cer | GM3(Gc) | - |
| 10 | NeuAcα-3Galß-4Glcß-Cer | Haematoside | - |
| 11 | NeuAcα-3Galß-4Glcß-Cer36 | GSC-17 | - |
| 12 | NeuAcα-3Galß-4Glc-DH | NeuAcα-(3')Lac | 1,660 |
| 13 | NeuAcα-3Galß-4Glc-AO | NeuAcα-(3')Lac-AO | - |
| 14 | NeuAcß-3Galß-4Glc-AO | NeuAcß-(3')Lac-AO | - |
| 15 | Neuα-3Galß-4Glc-AO | Neuα-(3')Lac-AO | - |
| 16 | 4O-AcNeuAcα-3Galß-4Glc-DH | Neu4,5Ac-(3')Lac | - |
| 17 | 4O-AcNeuAcα-3Galß-4Glc-AO | Neu4,5Ac-(3')Lac-AO | - |
| 18 | (4-deoxy)NeuAcα-3Galß-4Glcß-Cer36 | GSC-75 | - |
| 19 | (7-deoxy)NeuAcα-3Galß-4Glcß-Cer36 | GSC-76 | - |
| 20 | (8-deoxy)NeuAcα-3Galß-4Glcß-Cer36 | GSC-77 | - |
| 21 | (9-deoxy)NeuAcα-3Galß-4Glcß-Cer36 | GSC-51 | - |
| 22 | (4,8-deoxy)NeuAcα-3Galß-4Glcß-Cer36 | GSC-153 | - |
| 23 | (4-OMe)NeuAcα-3Galß-4Glcß-Cer36 | GSC-78 | - |
| 24 | (9-OMe)NeuAcα-3Galß-4Glcß-Cer36 | GSC-79 | - |
| 25 | KDNα-3Galβ-4Glcß-Cer28 | GSC-197 | - |
| 26 | KDNα-3Galβ-4Glcß-Cer34 | GSC-198 | - |
| 27 | NeuAcα-3Galß-3GlcNAc-DH | NeuAcα-(3')LN1-3 | - |
| 28 | NeuAcα-3Galß-3GlcNAc-AO | NeuAcα-(3')LN1-3-AO | - |
| 29 | NeuAcα-3Galß-4GlcNAc-DH | NeuAcα-(3')LN | 11,740 |
| 30 | NeuAcα-3Galß-4GlcNAc-AO | NeuAcα-(3')LN-AO | - |
| 31 | NeuAcα-3(6-NAc)Galß-4GlcNAc-DH | PI-1 | 2,482 |
| 32 | NeuAcα-3(6-NAc)Galß-4GlcNAc-AO | PI-1-AO | - |
| 33 | NeuAcα-3(6-NBz)Galß-4GlcNAc-DH | PI-2 | 12,327 |
| 34 | NeuAcα-3(6-NBz)Galß-4GlcNAc-AO | PI-2-AO | 421 |
| 35 | NeuAcα-3Galß-3GlcNAc-AO  │  Fucα-4 | SA(3')-Lea-Tri-AO | - |
| 36 | NeuAcα-3Galß-4GlcNAc-AO  │  Fucα-3 | SA(3')-Lex-Tri-AO | - |
| 37 | 9O-AcNeuAcα-3Galß-3GlcNAcß-C30  │  Fucα-4 | GSC-513 | - |
| 38 | 9O-AcNeuAcα-3Galß-4GlcNAcß-C30  │  Fucα-3 | GSC-511 | - |
|  | ***2-3 Sialyl***  ***Lacto-N-neotetraose and Lacto-N-tetraose-based linear backbones*** |  |  |
| 39 | NeuAcα-3Galß-3GlcNAcß-3Galß-4Glc-DH | LSTa | - |
| 40 | NeuGcα-3Galβ-3GlcNAcβ-3Galβ-4Glcß-C30 | GSC-396 | - |
| 41 | KDNα-3Galβ-3GlcNAcβ-3Galβ-4Glcß-Cer36 | GSC-147 | - |
| 42 | NeuAcα-3Galß-4GlcNAcß-3Galß-4Glcß-Cer | Sialylparagloboside | - |
| 43 | NeuAcα-3Galß-4GlcNAcß-3Galß-4Glcß-Cer36 | GSC-31 | - |
| 44 | Neuα-3Galß-4GlcNAcß-3Galß-4Glcß-Cer36  │  SU-6 | GSC-516B | - |
| 45 | NeuAcα-3Galβ-4GlcNAcβ-3Galβ-3GlcNAc-DH  │ │ │  SU-6 SU-6 SU-6 | C4U | - |
| 46 | NeuAcα-3Galß-3GlcNAcß-3Galß-4Glc-DH  │  Fucα-4 | SA(3')-LNFP-II | - |
| 47 | NeuAcα-3Galß-4GlcNAcß-3Galß-4Glc-DH  │  Fucα-3 | SA(3')-LNFP-III | - |
| 48 | NeuAcα-3Galß-4GlcNAcß-3Galß-Cer36  │  Fucα-3 | GSC-105 | - |
| 49 | NeuGcα-3Galβ-4GlcNAcβ-3Galß-Cer36   │   Fucα-3 | GSC-177 | - |
| 50 | KDNα-3Galβ-4GlcNAcβ-3Galß-C30  │   Fucα-3 | GSC-341 | - |
| 51 | NeuAcα-3Galß-4GlcNAcß-3Galß-4Glcß-Cer36  │  Fucα-3 | GSC-64 | - |
| 52 | KDNα-3Galβ-4GlcNAcβ-3Galβ-4Glcß-Cer36  │   Fucα-3 | GSC-149 | - |
| 53 | Neuα-3Galß-4GlcNAcß-3Galß-4Glcß-Cer36  │  Fucα-3 | GSC-472 | - |
| 54 | SU-6  │ NeuAcα-3Galß-4GlcNAcß-3Galß-4Glcß-Cer36  │  Fucα-3 | GSC-268 | - |
| 55 | SU-6  │ Neuα-3Galß-4GlcNß-3Galß-4Glcß-Cer36  │  Fucα-3 | GSC-268 deNAc | - |
| 56 | SU-6  │ NeuAcα-3Galß-4GlcNAcß-3Galß-4Glcß-Cer36  │  Fucα-3 | GSC-269 | - |
| 57 | SU-6  │ Neuα-3Galß-4GlcNAcß-3Galß-4Glcß-Cer36  │  Fucα-3 | GSC-406 | - |
| 58 | SU-6 SU-6  │ │ NeuAcα-3Galß-4GlcNAcß-3Galß-4Glcß-Cer36  │  Fucα-3 | GSC-270 | - |
| 59 | NeuAcα-3Galß-4GlcNAcß-3Galß-4GlcNAcß-3Galß-4Glcß-Cer36  │ │  Fucα-3 Fucα-3 | GSC-220 | - |
| 60 | NeuAcα-3Galß-4GlcNAcß-3Galß-4GlcNAcß-3Galß-4Glcß-Cer36  │  Fucα-3 | GSC-221 | - |
|  | ***2-3 Sialyl***  ***Branched backbone*** |  |  |
| 61 | Galß-4GlcNAcß-6  │ │  Fucα-3 Galß-4Glc-DH  │ NeuAcα-3Galß-3GlcNAcß-3 | MSMFLNH | - |
|  | ***2-3 Sialyl***  ***N-glycans*** |  |  |
| 62 | NeuAcα-3Galß-4GlcNAcß-2Manα-6 Fucα-6  │ │  Manß-4GlcNAcß-4GlcNAc-DH  │ NeuAcα-3Galß-4GlcNAcß-2Manα-3 | A2F(2-3) | - |
|  | ***2-3 Sialyl***  ***Complex ganglioside related*** |  |  |
| 63 | GalNAcβ-4Galβ-4Glcβ-Cer  │  NeuAcα-3 | GM2 | - |
| 64 | GalNAcβ-4Galβ-4Glcß-Cer36   │   KDNα-3 | GSC-193 | - |
| 65 | Galβ-3GalNAcβ-4Galβ-4Glcβ-Cer  │  NeuAcα-3 | GM1 | - |
| 66 | Galβ-3GalNAcβ-4Galβ-4Glc-DH  │  NeuAcα-3 | GM1-penta | - |
| 67 | Galβ-3GalNAcβ-4Galβ-4Glcβ-Cer  │  NeuGcα-3 | GM1(Gc) | - |
| 68 | Galβ-3GalNAcβ-4Galβ-4Glc-DH  │  NeuGcα-3 | GM1(Gc)-penta | - |
| 69 | KDNα-3Galβ-3GalNAcβ-4Galβ-4Glcß-Cer36   │   KDNα-3 | GSC-195 | - |
| 70 | NeuAcα-3Galß-3GalNAcß-4Galß-4Glcß-Cer  │  NeuAcα-3 | GD1a | - |
| 71 | NeuAcα-3Galß-3GalNAcß-4Galß-4Glc-DH  │  NeuAcα-3 | GD1a-hexa | - |
| 72 | GalNAcß-4Galß-3GalNAcß-4Galß-4Glcß-Cer   │ │  NeuGcα-3 NeuAcα-3  GalNAcß-4Galß-3GalNAcß-4Galß-4Glcß-Cer   │ │  NeuAcα-3 NeuGcα-3 | GalNAc-GD1a(Ac,Gc) | - |
|  | ***2-3 Sialyl***  ***Miscellaneous*** |  |  |
| 73 | NeuAcα-3Galß-4GlcNAcß-6Galß-4Glcß-Cer36  │  Fucα-3 | GSC-154 | - |
| 74 | NeuAcα-3Galß-4GlcNAcß-6GalNAcα-3Galß-4Glcß-C30 | GSC-441 | - |
|  | ***2-6 Sialyl***  ***Gal, Lactose and N-acetyllactosamine-based linear backbones*** |  |  |
| 75 | KDNα-6Galβ-Cer36 | GSC-144 | - |
| 76 | NeuAcα-6Galß-4Glcß-Cer36 | GSC-61 | - |
| 77 | NeuAcα-6Galß-4Glc-DH | NeuAcα-(6')Lac | - |
| 78 | NeuAcα-6Galß-4Glc-AO | NeuAcα-(6')Lac-AO | - |
| 79 | NeuAcß-6Galß-4Glc-AO | NeuAcß-(6')Lac-AO | - |
| 80 | Neuα-6Galß-4Glc-AO | Neuα-(6')Lac-AO | - |
| 81 | NeuAcα-6Galß-4GlcNAc-DH | NeuAcα-(6')LN | - |
| 82 | NeuAcα-6Galß-4GlcNAc-AO | NeuAcα-(6')LN-AO | - |
| 83 | 9O-AcNeuAcα-6Galß-4GlcNAc-DH | Neu5,9Ac-(6')LN | - |
|  | ***2-6 Sialyl***  ***Lacto-N-neotetraose and Lacto-N-tetraose-based linear backbones*** |  |  |
| 84 | Galβ-3GlcNAcβ-3Galβ-4Glc-DH  │ NeuAcα-6 | LSTb | - |
| 85 | NeuAcα-6Galβ4-GlcNAcβ3-Galβ4-Glc-DH | LSTc | - |
| 86 | NeuAcα-6Galß-4GlcNAcß-3Galß-4Glcß-Cer36  │  Fucα-3 | GSC-97 | - |
| 87 | NeuAcα-6Galß-4GlcNAcß-3Galß-4Glc-DH  │  Fucα-3 | SA(6')-LNFP-VI | - |
|  | ***2-6 Sialyl***  ***Branched backbone*** |  |  |
| 88 | NeuAcα-6Galß-4GlcNAcß-6  │  Galß-4Glc-DH  │  Galß-3GlcNAcß-3 | MSLNH | - |
| 89 | Galß-4GlcNAcß-6  │  Galß-4Glc-DH  │ NeuAcα-6Galß-3GlcNAcß-3 | MSLNnH-I | - |
| 90 | NeuAcα-6Galß-4GlcNAcß-6  │   Galß-4Glc-DH  │  NeuAcα-6Galß-4GlcNAcß-3 | DSLNnH | - |
| 91 | Galß-4GlcNAcß-6  │ │  Fucα-3 Galß-4Glc-DH  │ NeuAcα-6Galß-3GlcNAcß-3 | MFMSLNnH | - |
|  | ***2-6 Sialyl***  ***N-glycans*** |  |  |
| 92 | NeuAcα-6Galß-4GlcNAcß-2Manα-6  │   Manß-4GlcNAcß-4GlcNAc-DH  │ NeuAcα-6Galß-4GlcNAcß-2Manα-3 | A2(2-6) | - |
|  | ***2-6 Sialyl***  ***Ganglioside related*** |  |  |
| 93 | GalNAcβ-4Galβ-4Glcß-Cer36   │   NeuAcα-6 | GSC-442 | - |
| 94 | NeuAcα-6Galß-3GalNAcß-4Galß-4Glcß-Cer36 | GSC-68 | - |
| 95 | Galβ-3GalNAcβ-4Galβ-4Glcß-Cer36   │  NeuAcα-6 | GSC-155 | - |
| 96 | NeuAcα-6Galβ-3GalNAcβ-4Galβ-4Glcß-Cer36   │   NeuAcα-6 | GSC-107 | - |
|  | ***2-6 Sialyl***  ***Miscellaneous*** |  |  |
| 97 | NeuAcα-6Galß-6GalNAcß-4Galß-4Glcß-Cer36 | GSC-70 | - |
|  | ***2-3 and 2-6 Sialyl***  ***O-glycan related*** |  |  |
| 98 | NeuAcα-3Galß-3GalNAc-DH  │  NeuAcα-6 | DST | - |
|  | ***2-3 and 2-6 Sialyl***  ***Lacto-N-neotetraose and Lacto-N-tetraose-based linear backbone*** |  |  |
| 99 | NeuAcα-3Galß-3GlcNAcß-3Galß-4Glc-DH   │  NeuAcα-6 | DSLNT | - |
|  | ***2-3 and 2-6 Sialyl***  ***N-glycan*** |  |  |
| 100 | NeuAcα-3Galß-4GlcNAcß-2Manα-6  │   Manß-4GlcNAcß-4GlcNAc-DH  │ NeuAcα-3Galß-4GlcNAcß-4Manα-3  │  NeuAcα-6Galß-4GlcNAcß-2 | A3 | - |
|  | ***2-3 and 2-6 Sialyl***  ***Ganglioside related*** |  |  |
| 101 | NeuAcα-3Galβ-3GalNAcβ-4Galβ-4Glcß-Cer36   │   NeuAcα-6 | GSC-118 | - |
|  | ***2-8 Sialyl***  ***Ganglioside related*** |  | - |
| 102 | NeuAcα-8NeuAcα-3Galß-4Glcß-Cer | GD3 | - |
| 103 | NeuAcα-8NeuAcα-3Galß-4Glc-DH | GD3-tetra | - |
| 104 | NeuAcα-8NeuAcα-3Galß-4Glc-AO | GD3-tetra-AO | - |
| 105 | NeuAcα-8NeuAcα-8NeuAcα-3Galβ-4Glcß-Cer36 | GSC-437 | - |
| 106 | GalNAcß-4Galß-4Glcß-Cer  │ NeuAcα-8NeuAcα-3 | GD2 | - |
| 107 | Galß-3GalNAcß-4Galß-4Glcß-Cer  │ NeuAcα-8NeuAcα-3 | GD1b | - |
| 108 | NeuAcα-8NeuAcα-3Galβ-3GalNAcβ-4Galβ-4Glcβ-Cer  │  NeuAcα-8NeuAcα-3 | GQ1b | - |
|  | ***2-8 Sialyl***  ***Polysialyl*** |  |  |
| 109 | NeuAcα-8NeuAc-DH | SA2(α8) | - |
| 110 | NeuAcα-8NeuAcα-8NeuAc-DH | SA3(α8) | - |
| 111 | NeuAcα-8NeuAcα-8NeuAcα-8NeuAc-DH | SA4(α8) | - |
| 112 | NeuAcα-8NeuAcα-8NeuAcα-8NeuAcα-8NeuAc-DH^C^ | SA5(α8) | - |
| 113 | NeuAcα-8NeuAcα-8NeuAcα-8NeuAcα-8NeuAcα-8NeuAc-DH^C^ | SA6(α8) | - |
| 114 | NeuAcα-8NeuAcα-8NeuAcα-8NeuAcα-8NeuAcα-8NeuAcα-8NeuAc-DH^C^ | SA7(α8) | - |
| 115 | NeuAcα-8NeuAcα-8NeuAcα-8NeuAcα-8NeuAcα-8NeuAc-8NeuAcα-8NeuAc-DH^C^ | SA8(α8) | - |
| 116 | NeuAcα-8NeuAcα-8NeuAcα-8NeuAcα-8NeuAcα-8NeuAc-8NeuAcα-8NeuAc-8NeuAcα-DH^C^ | SA9(α8) | - |
| 117 | NeuAcα-8NeuAcα-8NeuAcα-8NeuAcα-8NeuAcα-8NeuAc-8NeuAcα-8NeuAcα-8NeuAcα-8NeuAc-DH^C^ | SA10(α8) | - |
| 118 | NeuAcα-8NeuAcα-8NeuAcα-8NeuAcα-8NeuAcα-8NeuAc-8NeuAcα-8NeuAαc-8NeuAcα-8NeuAcα-8NeuAc-DH^C^ | SA11(α8) | - |
|  | ***2-3 and 2-8 Sialyl***  ***Ganglioside related*** |  |  |
| 119 | NeuAcα-8NeuAcα-3Galβ-3GalNAcβ-4Galβ-4Glcβ-Cer  │  NeuAcα-3 | GT1a | - |
| 120 | NeuAcα-3Galβ-3GalNAcβ-4Galβ-4Glcβ-Cer  │  NeuAcα-8NeuAcα-3 | GT1b | - |
|  | ***2-9 Sialyl***  ***Gal, Lactose and N-acetyllactosamine-based linear backbones*** |  |  |
| 121 | NeuAcα-9NeuAcα-3Galβ-4Glcß-Cer36 | GSC-96 | - |
|  | ***Monosaccharides*** |  |  |
| 122 | NeuAc-AO | NeuAc-AO | - |
| 123 | NeuGc-AO | NeuGc-AO | - |

^a^ The oligosaccharide probes are all lipid-linked, and are from the collection assembled in the course of research in Glycosciences Laboratory. DH, designates NGLs prepared from reducing oligosaccharides by reductive amination with the amino lipid, 1,2-dihexadecyl-*sn*-glycero-3-phosphoethanolamine (DHPE) (1); AO, NGLs prepared from reducing oligosaccharides by oxime ligation with an aminooxy-functionalized DHPE (2); Cer, natural glycolipids with various ceramide moieties; GSC,designations for synthetic glycolipids in Kiso laboratory; Cer36, synthetic glycolipids with ceramide having a total of 36 carbon atoms; C30, a synthetic lipid [2-(tetradecyl)hexadecanol] with 30 carbon atoms. NB, Neu denotes de-N-acetylated neuraminic acid.

^b^ -, Signal less than 1.

^c^ Major component.

1. Chai, W., Stoll, M. S., Galustian, C., Lawson, A. M. & Feizi, T. Neoglycolipid technology - deciphering information content of glycome. *Methods Enzymol.* **362**, 160-195 (2003).

2. Liu, Y. *et al.* Neoglycolipid probes prepared via oxime ligation for microarray analysis of oligosaccharide-protein interactions. *Chem. Biol.* **14**, 847-859 (2007).
